# Investigation of hub gene associated with the infection of *Staphylococcus aureus* via weighted gene co-expression network analysis

**DOI:** 10.1186/s12866-021-02392-y

**Published:** 2021-12-01

**Authors:** Jia-xin Li, Xun-jie Cao, Yuan-yi Huang, Ya-ping Li, Zi-yuan Yu, Min Lin, Qiu-ying Li, Ji-chun Chen, Xu-guang Guo

**Affiliations:** 1 Department of Clinical Laboratory Medicine, The Third Affiliated Hospital of Guangzhou Medical University, Guangzhou, 510150 China; 2 Department of Clinical Medicine, The First Clinical School of Guangzhou Medical University, Guangzhou, 511436 China; 3 Department of Clinical Medicine, The Third Clinical School of Guangzhou Medical University, Guangzhou, 511436 China; 4 Department of Clinical Medicine, The Second Clinical School of Guangzhou Medical University, Guangzhou, 511436 China; 5 Department of Traditional Chinese and Western Clinical Medicine, The Traditional Chinese and Western Clinical School of Guangzhou Medical University, Guangzhou, 511436 China; 6 Key Laboratory for Major Obstetric Diseases of Guangdong Province, The Third Affiliated Hospital of Guangzhou Medical University, Guangzhou, 510150 China

**Keywords:** *Staphylococcus aureus*, Weighted gene co-expression network analysis, Mitophagy, Bioinformatic analysis

## Abstract

**Introduction:**

*Staphylococcus aureus* is a gram-positive bacterium that causes serious infection. With the increasing resistance of bacteria to current antibiotics, it is necessary to learn more about the molecular mechanism and cellular pathways involved in the *Staphylococcus aureus* infection.

**Methods:**

We downloaded the GSE33341 dataset from the GEO database and applied the weighted gene co-expression network analysis (WGCNA), from which we obtained some critical modules. Kyoto Encyclopedia of Genes and Genomes (KEGG) and Gene Ontology (GO) were applied to illustrate the biological functions of genes in these modules. We constructed the protein-protein interaction (PPI) network by Cytoscape and selected five candidate hub genes. Five potential hub genes were validated in GSE30119 by GraphPad Prism 8.0. The diagnostic values of these genes were calculated and present in the ROC curve based on the GSE13670 dataset. Their gene functions were analyzed by Gene Set Enrichment Analysis (GSEA).

**Results:**

A co-expression network was built with 5000 genes divided into 11 modules. The genes in green and turquoise modules demonstrated a high correlation. According to the KEGG and GO analyses, genes in the green module were closely related to ubiquitination and autophagy. Subsequently, we picked out the top five hub genes in the green module. And UBB was determined as the hub gene in the GSE30119 dataset. The expression level of UBB, ASB, and MKRN1 could significantly differentiate between *Staphylococcus aureus* infection and healthy controls based on the ROC curve. The GSEA analysis indicated that lower expression levels of UBB were associated with the P53 signal pathway.

**Conclusions:**

We identified some hub genes and significant signal enrichment pathways in *Staphylococcus aureus* infection via bioinformatics analysis, which may facilitate the development of potential clinical therapeutic strategies.

**Supplementary Information:**

The online version contains supplementary material available at 10.1186/s12866-021-02392-y.

## Introduction

As a gram-positive bacterium, [1–3] *Staphylococcus aureus* can infect various tissue and organs in human beings, causing mild skin infection or even severe illnesses [4]. It causes conditions such as endocarditis, osteomyelitis, septicemia, and toxic shock syndrome toxin (TSST), [5–7] primarily through the production of a variety of toxic factors and toxins [5, 6]. Though most of the antibiotics used to treat *Staphylococcus aureus* work well, emerging evidence has shown that *Staphylococcus aureus*’s resistance becomes a major issue for clinics [2, 6]. For instance, the methicillin-resistant *S. aureus* (MRSA), [8, 9] one of the most common resistant strains, leads to increased mortality and morbidity, which makes it difficult for clinicians to prescribe the suitable treatment therapy [1, 10]. By forming colonies in human nares and skin, *Staphylococcus aureus* causes mild symptoms while invading deeper tissues and exposed organs in a vulnerable environment, which causes severe diseases [2, 6]. Given the lack of effective treatment regimens and vaccines for drug-resistant *Staphylococcus aureus*, [11, 12] it is necessary to explore potential target genes associated with *Staphylococcus aureus*.

In this study, we construct a gene co-expression network using the WGCNA method and select some modules that pique our interest for further analysis. KEGG and GO analyses were applied to explore the biological functions of the modules. We obtained five hub genes through STRING and Cytoscape and explored the relationship between these genes and *Staphylococcus aureus* infection prognosis through the ROC curve. Moreover, we further addressed the biological roles of these genes during infection through GSEA. These results offer some critical genetic candidates for treating *Staphylococcus aureus* infection, which may facilitate vaccine development and treatment optimization for *Staphylococcus aureus* infection.

## Methods and materials

### Data inclusion and processing

**Fig. 1:**
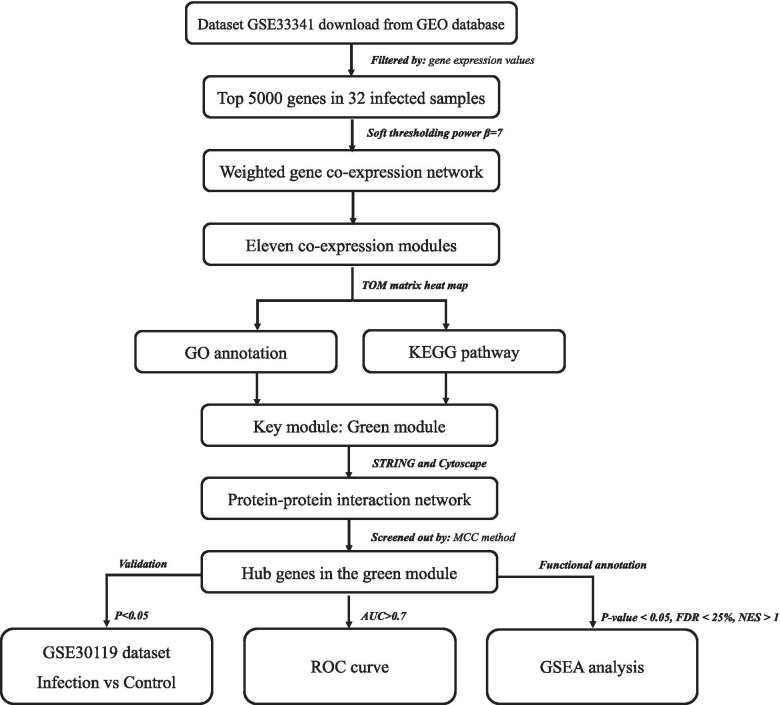
Workflow of the study. Fig. 1 displayed data preparation, processing, and analysis in this study

Figure 1 displayed the workflow in our study. The data processing methods applied in our study were carried out in accordance with relevant guidelines and regulations. The Gene Expression Omnibus (GEO) is a comprehensive database of gene expression, collecting microarray and high-throughput resources. (http://www.ncbi.nlm.nih.gov/geo/). In this study, we downloaded the gene expression profile of GSE33341 [13] from the GEO database. The GSE33341 dataset was based on the GPL1261 platform of Affymetrix Mouse Genome 430 2.0 Array and GPL571 platform of Affymetrix Human Genome U133A 2.0 Array. We extracted the data that included 32 human samples infected with *Staphylococcus aureus* and 43 healthy counterparts. Then, we converted gene probes into gene symbols based on microarray annotation information on the GPL571 platform. For probes corresponding to multiple gene symbols, we randomly selected one gene symbol for matching the probes. Probes with no corresponding gene symbols were removed. For gene symbols corresponding to multiple probes, we reserved the probe with the highest average value. Through the above steps, we ensure the one-to-one correspondence between probes and gene symbols. Furthermore, genes with negative values were deleted.

### Weighted gene co-expression network analysis

**Fig. 2:**
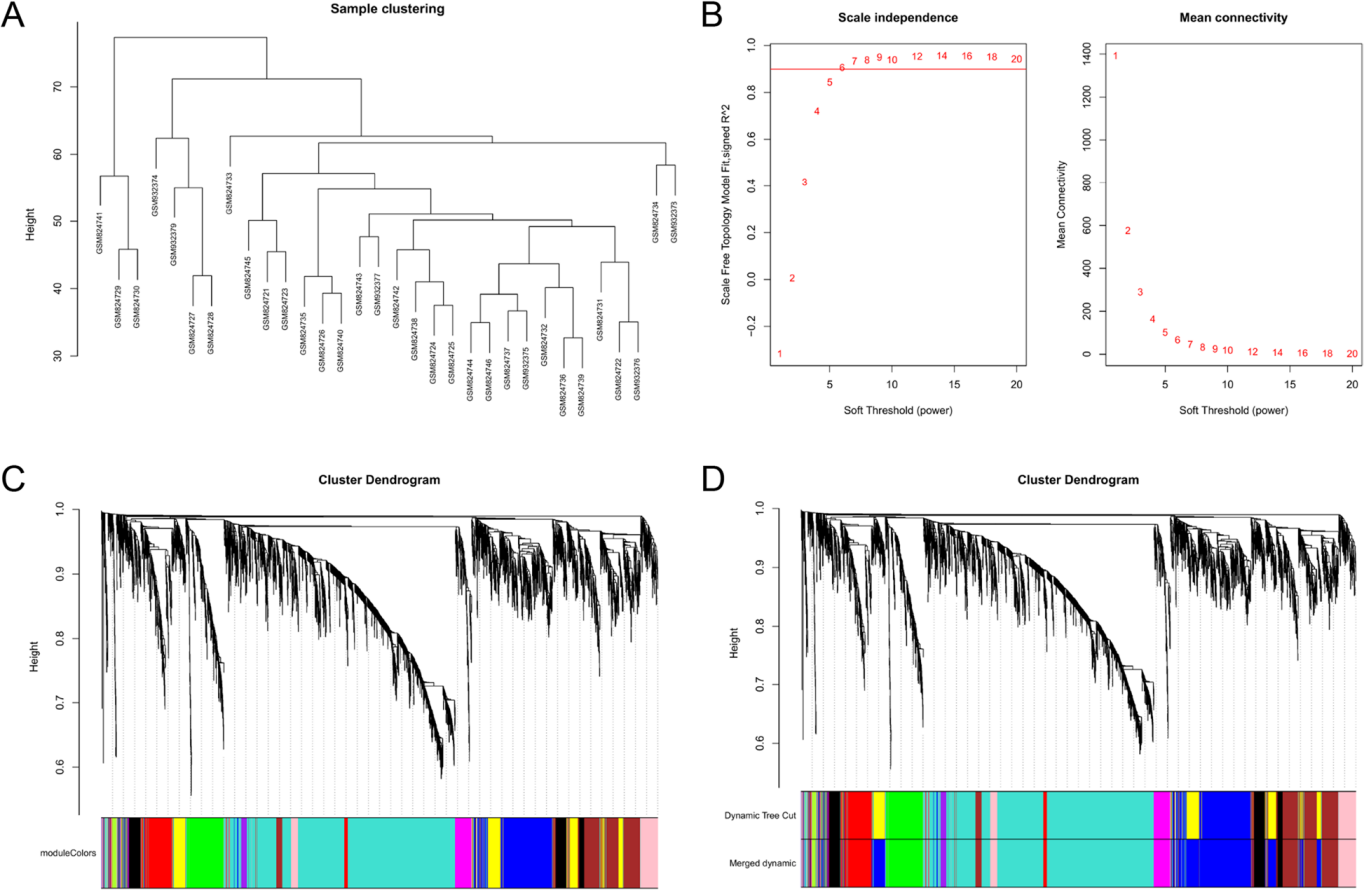
Construction of co-expression modules for *Staphylococcus aureus* by WGCNA. (**A**) Clustering of samples in GSE33341 to identify outliers. There is no obvious outlier that needed to remove. (**B**) Determination of soft-thresholding power in WGCNA analysis. The figure showed the scale-free fitting index (left) and average connectivity (right) corresponding to different soft thresholds. And soft thresholding power selected was 7. (**C**) The cluster Dendrogram of 5000 genes . Each branch represents a gene and the different colors below represent different modules. A total of 11 co-expression modules were constructed. (**D**) The cluster Dendrogram after merging. When setting the height cutoff value as 0.4, the blue and yellow modules with high correlation are merged. Eventually, there were ten modules in total

The top 5000 genes with high expression values in 32 infected samples were selected to construct co-expression modules through the R package “WGCNA”.(Fig. 2A) WGCNA is a systems biology approach applied to search for highly correlated gene modules or identify biomarkers for candidate diseases [14]. When the soft thresholding power β was 7, the scale-free R^2^ of the co-expression network was close to 0.9. (Fig. 2B). Therefore, network construction and module detection were continued based on the soft thresholding power β. To enhance the reliability of the results, we set the minimum number of genes in modules as 30. We further defined 0.4 as the threshold for cut height to merge modules with high correlation.

### Functional and pathway enrichment analyses of genes in the critical modules

WGCNA can divide genes with similar functions into the same module. Therefore, genes in the same co-expression modules possess a higher degree of connectivity, which means they may play similar roles. According to the TOM matrix heat map, we identified two key modules that were significantly associated with *Staphylococcus aureus* infection.

KEGG is an integrated database for analyzing genomes and biological data [15–17]. (www.kegg.jp/kegg/kegg1.html) Gene ontology (GO) is an international standardized classification system for gene function, covering biological process, molecular function, and cellular components [18]. To study the biological function of the genes in the important modules, we performed KEGG and GO pathway enrichment analyses by R language. *P* < 0.05 was set as the cut-off value.

### Hub genes identification

PPI network is a useful tool to understand cell functions and disease machinery, which is crucial in predicting the function of interacting proteins [19]. We imported the genes previously obtained from the critical modules into the online database STRING (version 11.0; https://string-db.org/), which helps constructing the PPI network. The combined score was set as over 0.4. Subsequently, we searched the key modules and hub genes in the PPI network by the MCODE plugin and CytoHubba plugin in Cytoscape software. Finally, we defined the top five genes that displayed the highest degree of connectivity in key modules as hub genes.

### Validation of hub genes in datasets

To ensure the result’s rigor, we used two other datasets, GSE30119 [20] and GSE13670 [21], to verify the hub genes. In the GSE30119 dataset, the data of 99 infected samples and 44 healthy samples were extracted from whole blood and imported into GraphPad Prism (version 8.0.2) for t-tests and non-parametric tests. The hub genes with *P* < 0.05 were considered significant. In the GSE13670 dataset, 15 infected blood samples and 15 healthy samples were utilized to plot ROC curves, from which we obtained their AUC through the “pROC” package. ROC curve is usually a helpful tool to evaluate the efficiency of gene diagnosis [22]. The hub genes with AUC > 0.7 were deemed useful for disease diagnosis.

### Gene set enrichment analysis

GSEA is an analytical method concentrating on groups of genes that share common biological functions and regulation [23]. To learn about the biological function, we performed the GSEA for the hub gene. Based on the median expression level of the hub gene, 32 infection samples were classified into the low-expression and the high-expression group. GSEA 4.1.0 was used to perform the analysis, and the c2.cp.kegg.v7.2.symbols.gmt in the Molecular Signatures Database (MSigDB) was selected as the reference gene set. The results met nominal *p*-value < 0.05, FDR < 25% and normalized enrichment score > 1 were considered statistically significant.

## Results

### Gene co-expression network construction and significant module identification

**Fig. 3:**
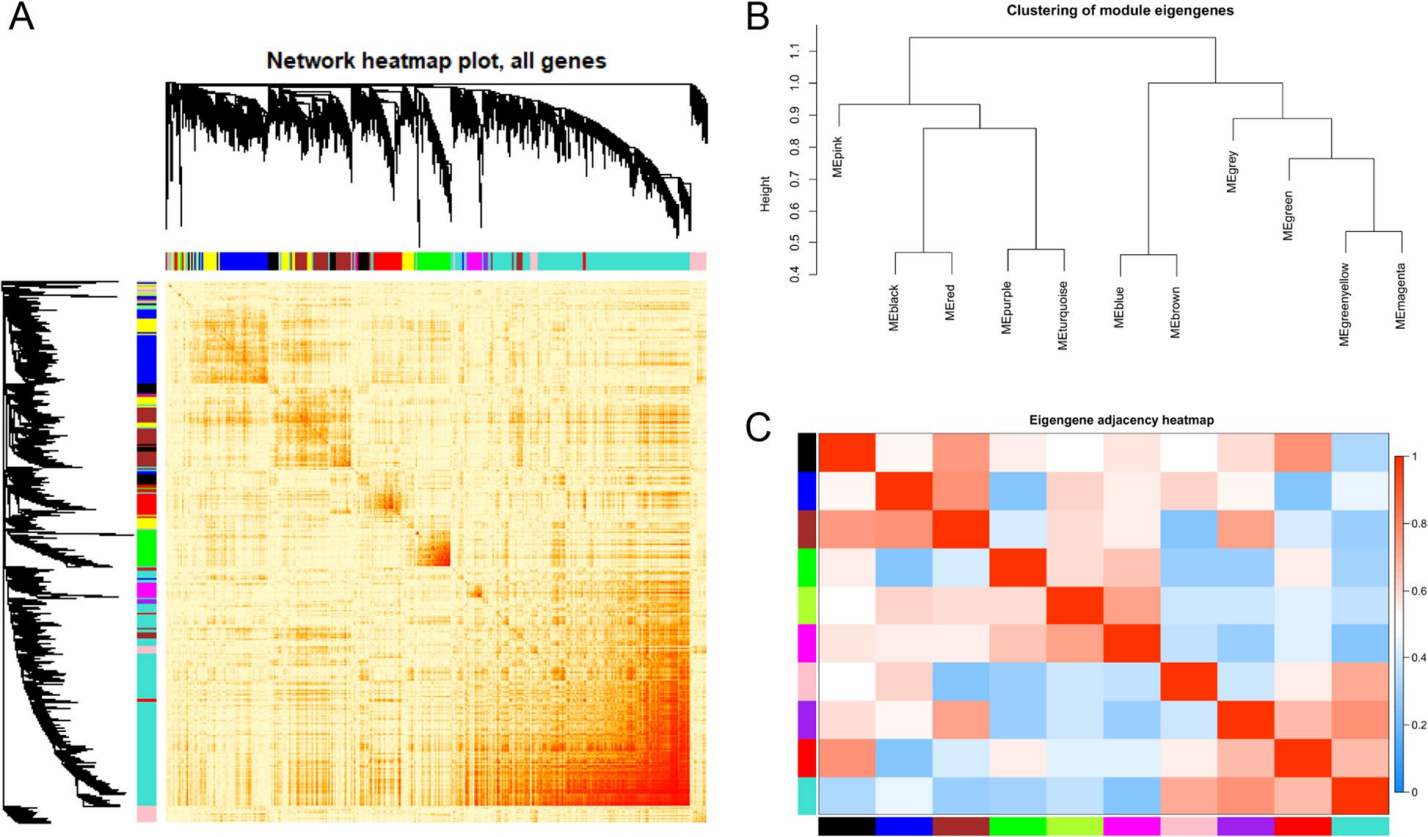
Key module identified by WGCNA. (**A**) Interaction relationships between genes in the co-expression modules. The brightness of yellow in the middle represents the correlation between the various modules. The figure showed that there were significant differences in the correlation among different modules. And red revealed that genes in the same module have closer relationships. (**B**) Hierarchical clustering dendrogram of the eigengenes. (**C**) Heatmap of the eigengene adjacencies. The depth of the color represents the connectivity of critical genes between different modules. And the red indicated a positive correlation while the blue indicated a negative correlation

After preliminary screening, we screened out 13, 345 genes from 32 infection samples to undergo WGCNA. The top 5000 genes with high expression values were screened out and used to construct the co-expression network. Through WGCNA analysis, 5000 genes were divided into 12 co-expression modules (Fig. 2C). Since yellow and blue modules had a high similarity, they were merged when MEDissThres was setting as 0.4. (Additional file 1) Therefore, there were a total of 11 modules, eventually (Fig. 2D). The turquoise module is the most extensive module and includes 1952 genes. The number of genes in the green-yellow module was the lowest, which only comprised 39 genes. A total of 92 genes that did not belong to any of the ten modules were classified as grey module. Next, we analyzed the interactions between genes in the co-expression modules. The results (Fig. 3A) revealed some noticeable differences in the correlation among other modules, especially the turquoise and green module, which aroused our attention and interest for further research. Moreover, we analyzed the connection between different co-expression modules by calculating the connectivity of eigengenes. As shown in Fig. 3B, 11 modules were divided into two clusters. One comprised pink, black, red, purple, and turquoise modules; the other included blue, brown, grey, green, green-yellow, and magenta modules. We observed that there were higher adjacencies between several pairs of modules from Fig. 3C, such as the brown and black, brown and blue, red and black, turquoise and purple modules.

### Functional enrichment analysis of genes in modules of interest

**Fig. 4:**
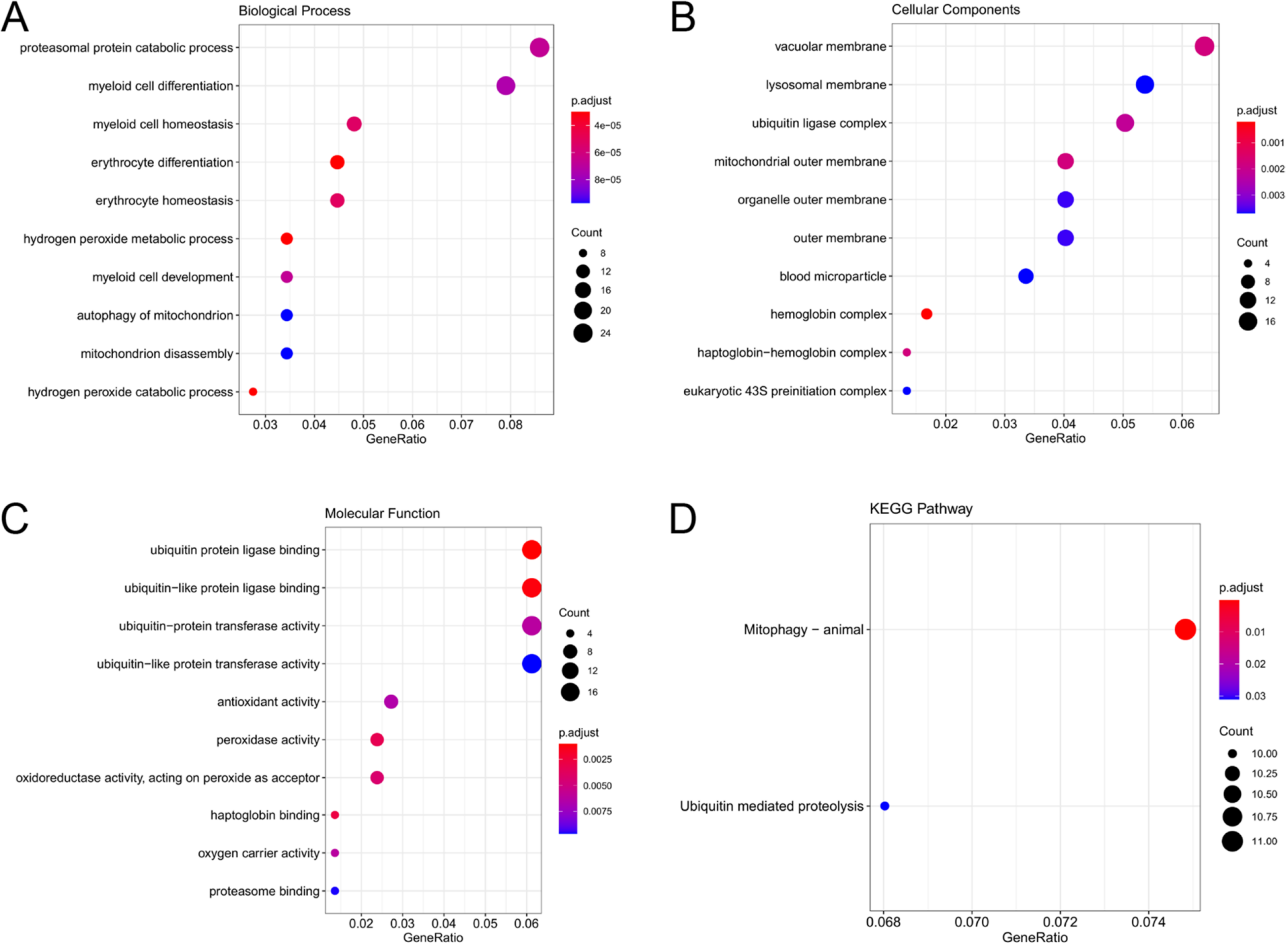
KEGG and GO analysis of the genes in the green module. (**A**) Enriched KEGG pathways of the green module. (**B**) GO enrichment of green module in Biological Process terms. (**C**) GO enrichment of green module in Molecular Function. (**D**) GO enrichment of green module in Cellular Component. The dot sizes represent the number of the enriched genes in the corresponding GO term, and the colors indicate the adjusted *P*-value

KEGG and GO were applied to obtain biological functions for the genes in the green and turquoise modules. According to the KEGG and GO enrichment analysis, the green module was defined as the critical module to undergo further analysis. The results of KEGG and GO analyses of the turquoise module were displayed in Additional file 2. Based on the KEGG analysis, mitophagy—animal and ubiquitin-mediated proteolysis were the most enriched terms in the green module (Fig. 4A). In terms of biological pathways, genes in the green module were enriched in the proteasomal protein catabolic process and myeloid cell differentiation catabolic process. (Fig. 4B) Concerning MF, genes were significantly enriched in ubiquitin-protein ligase binding (Fig. 4C). For CC, genes were involved in the vacuolar membrane. (Fig. 4D)

### PPI network analysis and hub genes identification

**Fig. 5:**
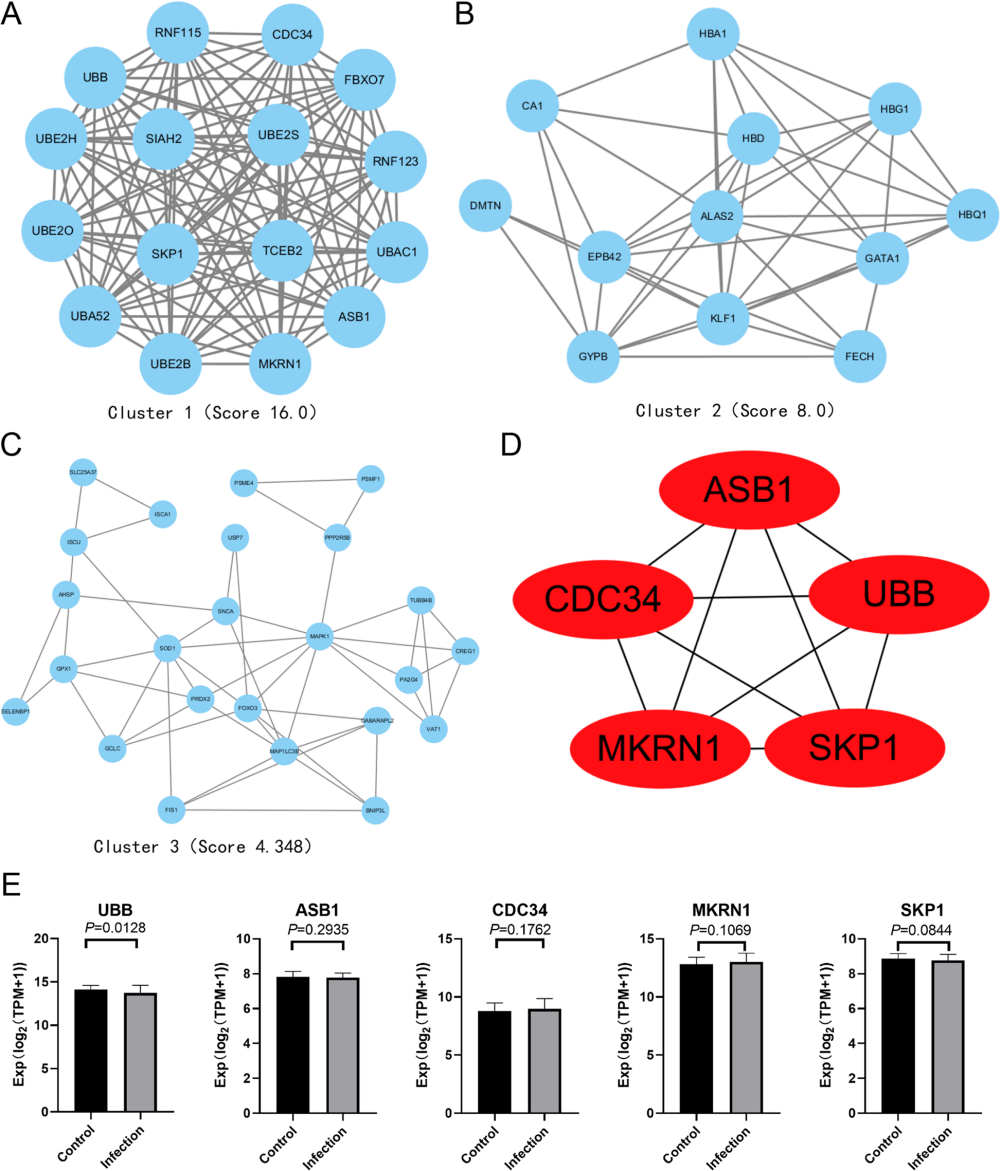
Hub genes identification and validation. (**A**-**C**) The modules extracted from the PPI network by the MCODE plugin. (**D**) The hub genes with the highest MCC score in the key module. (**E**) The expression of the hub genes in control and infection group

We built the PPI network of genes in the green module with the help of STRING. (Additional file 3) And we identified the dense regions among the PPI network through the MCODE plugin. (Fig. 5A, B, C) Finally, we picked out the significant module with the highest score (score: 16), 16 nodes, and 120 edges from the green module (Fig. 5A). The genes with the top five MCC values in the final module were defined as hub genes(ASB1, CDC34, SKP1, MKRN1, and UBB) (Fig. 5D).

### Verification and efficacy evaluation of hub genes

**Fig. 6:**
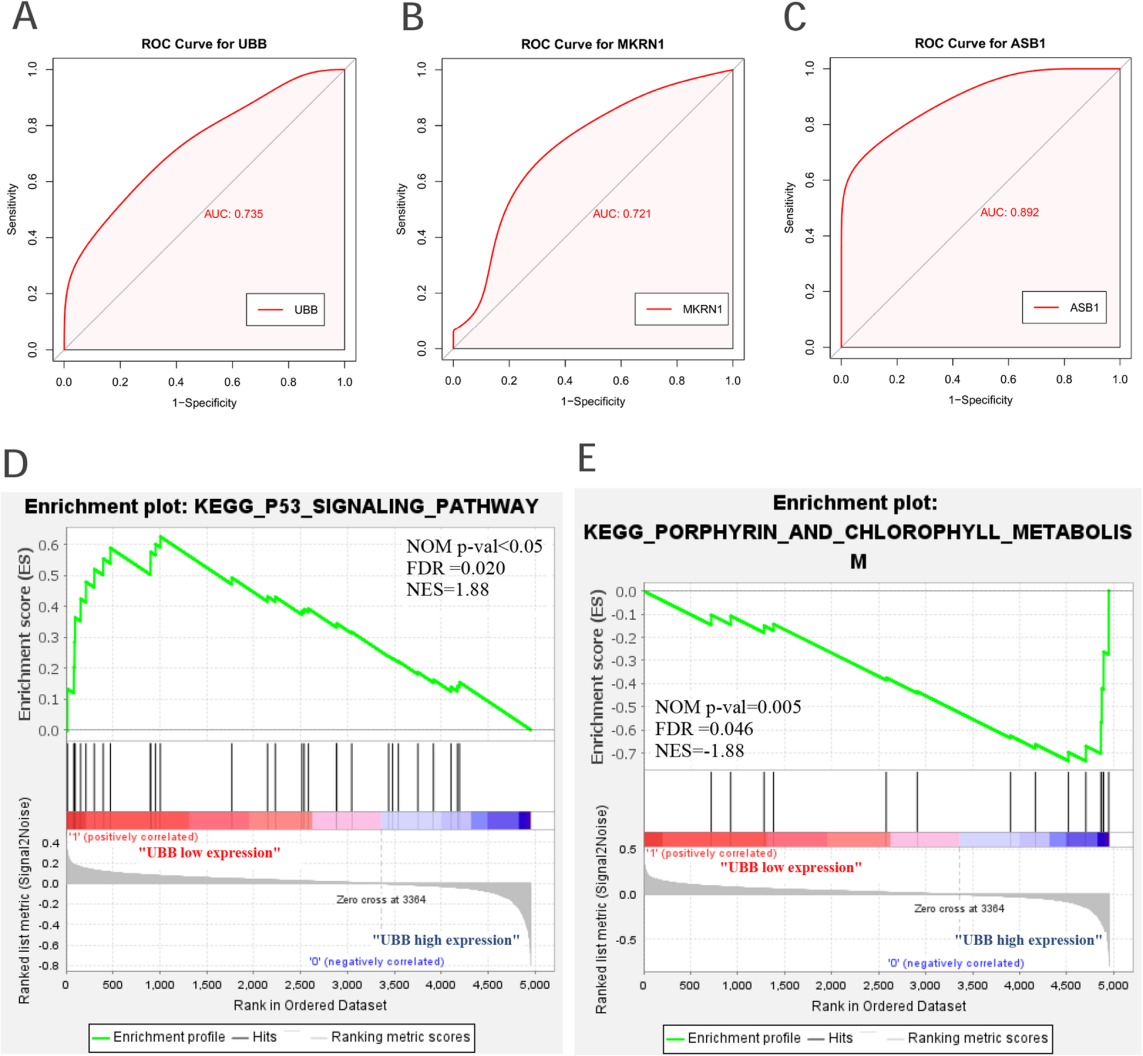
Diagnostic significance ability prediction and Gene Set Enrichment Analysis of UBB. (**A**, **B**, **C**) ROC curve of hub genes including ASB1, UBB, and MKRN1. The area under the ROC curve (AUC) for each gene displayed its accuracy for differentiation of Staphylococcus aureus infection and healthy subjects about sensitivity and specificity. (**D**, **E**) The enriched GSEA terms with significant statistics of UBB. Based on the normalized enrichment scores, the top one GSEA enrichment terms in the high and low expression group of UBB

In the GSE30119 dataset, with the standard of *P* < 0.05, UBB was defined as the hub gene (Fig. 5E). In the GSE13670 dataset, the area under the curve (AUC) of ASB1, UBB, and MKRN1 were both greater than 0.7, which suggested their potential diagnostic significance (Fig. 6A, B, C, and Additional file 4). In conclusion, we defined UBB as our hub gene to conduct further analysis in our study.

### Gene set enrichment analysis

After the GSEA for the final selected hub genes, we found that the KEGG P53 signaling pathway was associated with the samples with lower expression of UBB while KEGG porphyrin and chlorophyll metabolism was associated with the samples with higher expression of UBB (Fig. 6D and E).

## Discussion

Multiple drug-resistant *Staphylococcus aureus* has become a public health issue, which renders the clinical treatment of the infection difficult [24]. We aimed to analyze the RNA transcriptional profiles and explore the highly correlated genes that participate in *Staphylococcus aureus* infection. In this study, a total of 5000 differentially expressed genes were constructed into 11 modules by WGCNA. Based on this, we applied bioinformatics methods to explore the characteristics of *Staphylococcus aureus* infection.

Through WGCNA analysis, we defined the green module as the key module and the genes in the green module were extracted for analysis. According to KEGG analysis, mitophagy and ubiquitin-mediated proteolysis were the most significant enrichment results of the genes in the green module, both of which were closely associated with cell defense. Mitophagy is a metabolic process that participates in the removal of excessive or damaged mitochondria in eukaryotic cells, thus maintaining the stability of the intracellular microenvironment [25]. The previous study [26, 27] has proven that systemic sepsis or pneumonia caused by *Staphylococcus aureus* is associated with mitochondrial lung damage. Mitophagy is a strategy adopted by cells to eliminate damaged mitochondria and prevent their harmful byproducts, such as reactive oxygen species (ROS), from interfering with normal cell components like DNA and lipids [26, 28].

According to the GO analysis, the proteasomal protein catabolic process was one of the significant enrichment items in biological process. Proteasomal degradation was a crucial catabolic pathway in cells. And the up-regulation of this process contributes to activating the adaptive immune system and eliminating intracellular pathogens [29]. In terms of molecular function, genes in the green module were also significantly enriched in ubiquitin–protein ligase binding, ubiquitin−like protein ligase binding, ubiquitin−protein transferase activity, and ubiquitin-like protein transferase activity. These all had a close connection with the ubiquitination process. Ubiquitination is a sequential process in which ubiquitin binds with target proteins via enzymatic cascade and degrades them through proteasome or lysosome pathways [30, 31]. It plays an essential role in the regulation of immune responses and the defense against pathogens [32]. For example, Neumann, Yvonne, and Sakowski Erik reported that intracellular *Staphylococcus aureus* was ubiquitinated by the host cell shortly after the invasion and confused with lysosomes [33, 34]. In addition, ubiquitination is crucial in mitophagy. Damaged mitochondria were eliminated by binding to the ubiquitin ligase parkin [35].

Therefore, combining with KEGG, BP, and MF analysis, we concluded that the up-regulation of the protein ubiquitination pathway and mitophagy might help defend against *Staphylococcus aureus* infection.

Concerning cellular components, the vacuolar membrane is the most notable enrichment result. Some studies reported that *Staphylococcus aureus* can survive phagocytosis by macrophages and neutrophils and replicate in the cell as a vacuolar pathogen [36, 37]. In addition, the vacuolar pathogen can escape from vacuolar and replicate in the cytosol by modifying the vacuolar membranes, leading to a vicious cycle of host phagocytosis, host cell death, and bacterial release [36]. Therefore, we inferred that block enzymes that modify vacuolar membranes, such as phospholipases, can help prevent bacterial escape.

Protein-protein interactions are crucial for knowing about biological processes in living cells [38]. We selected the top-five critical genes from the PPI network to investigate their biological function in *Staphylococcus aureus* infection. According to the ROC curve, ASB1, UBB, and MKRN1 can effectively act as potential diagnostic markers for distinguishing samples of *Staphylococcus aureus* infection from healthy counterparts. ASB1, a member of the ASB family, acted as a positive regulator of NF-κB– and MAPK-mediated inflammatory signaling pathways [39]. Appropriate inflammatory response helps immune cells fight microbial infections while excessive inflammatory response can damage tissues and cells [40]. The study pointed that ASB1 deficiency protected mice from LPS- or bacteria-induced death by inhibiting inflammation [39]. Therefore, we speculated that inhibition and down-regulation of ASB1 in the late stage of infection can against the inflammatory injury induced by *Staphylococcus aureus*. Makorin ring finger protein 1 (MKRN1) is a ubiquitin ligase, which is vital against viral pathogens [41]. Some studies displayed that the high expression of MKRN1 can help host cells to defend against infection by inducing ubiquitination of pparγ that is beneficial to *M. tuberculosis* growth [41]. However, there are few studies about the role of MKRN1 in the *Staphylococcus aureus*. Through the verification of the dataset GSE30119, UBB was also identified as a hub gene. UBB was known to be a vital gene that encodes ubiquitin, one of the most conserved proteins that play a significant role in targeting cellular proteins for degradation by the 26S proteasome [42]. GSEA results revealed that the group with a low expression of UBB was closely associated with the p53 signaling pathway in *Staphylococcus aureus* infection. P53 is well known for its anti-cancer function and regulation of autophagy [43–45]. Abundant evidence revealed that cytosolic p53 could also disturb the process of mitophagy through inhibitory interaction with Parkin, a ubiquitin E3 ligase [43, 44] Zhang, Fei pointed out that the upregulation of p53 inhibited mitochondrial translocation of Parkin and activation of Parkin’s E3 ubiquitin ligase, which eventually stopped cells from effectively removing damaged mitochondria [46]. Therefore, we speculate that this was a strategy for bacteria to avoid death.

In this study, we found that ubiquitination and mitophagy play important roles in defending against *Staphylococcus aureus* infection. In addition, we also identified some hub genes which act as regulators in *Staphylococcus aureus* infection. However, more molecular biological experiments will be needed to confirm the function of the identified genes.

## Supplementary Information

**Additional file 1.**

**Additional file 2.**

**Additional file 3.**

**Additional file 4.**

## Data Availability

Microarray datasets (GSE33341, GSE13670 and GSE30119) for this study are openly available in Gene Expression Omnibus database at https://www.ncbi.nlm.nih.gov/geo/query/acc.cgi?acc=GSE33341, https://www.ncbi.nlm.nih.gov/geo/query/acc.cgi?acc=GSE13670, and https://www.ncbi.nlm.nih.gov/geo/query/acc.cgi?acc=GSE30119, respectively.
