# Supplementary figures and images for: Investigation of hub gene associated with the infection of Staphylococcus aureus via weighted gene co-expression network analysis
Source: BMC Microbiol. 2021 Dec 1;21:329. doi: 10.1186/s12866-021-02392-y (PMC8633612; doi:10.1186/s12866-021-02392-y)

# Clustering of module eigengenes

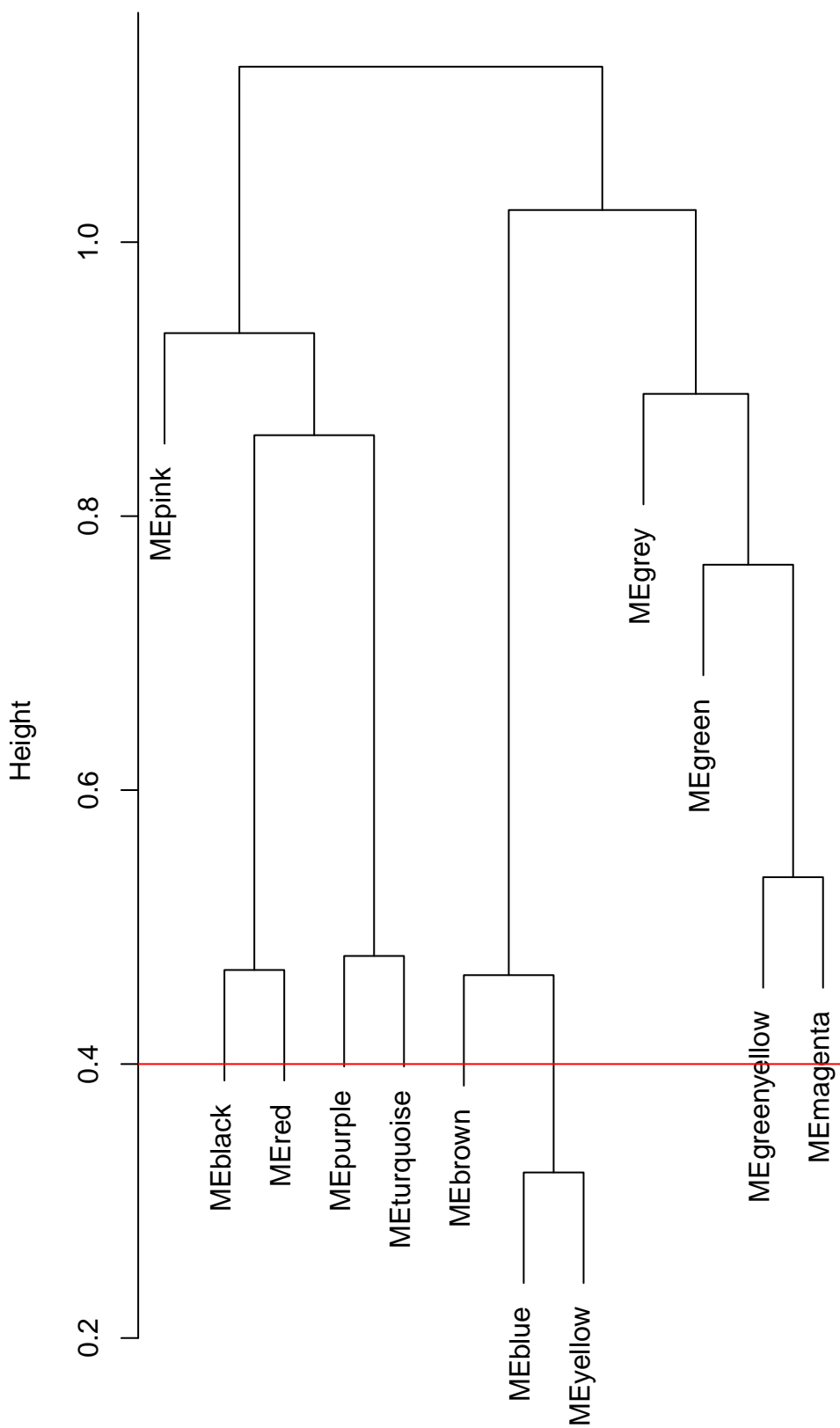

Supplement: Supplementary file 1 — Additional file 1. [file 12866_2021_2392_MOESM1_ESM.pdf]

A

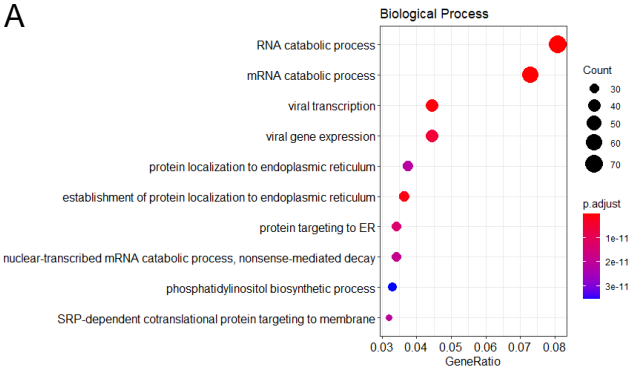

B

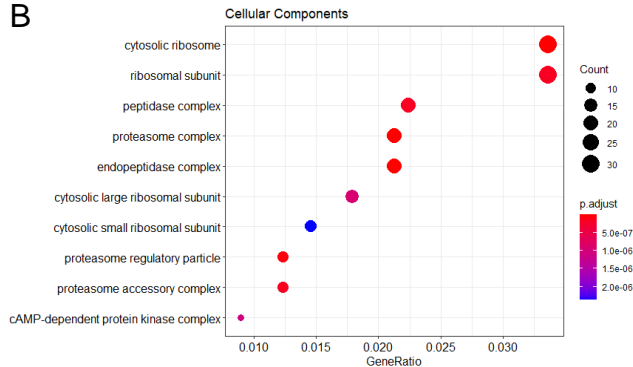

C

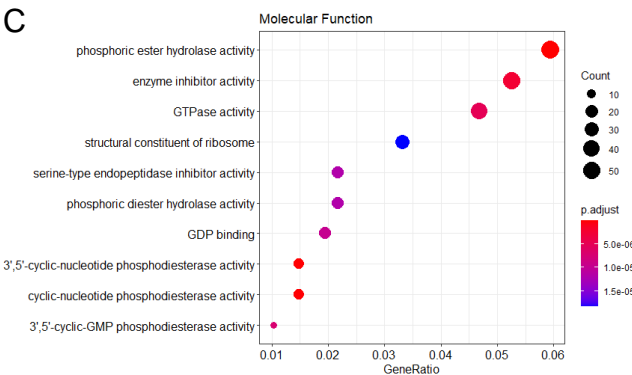

D

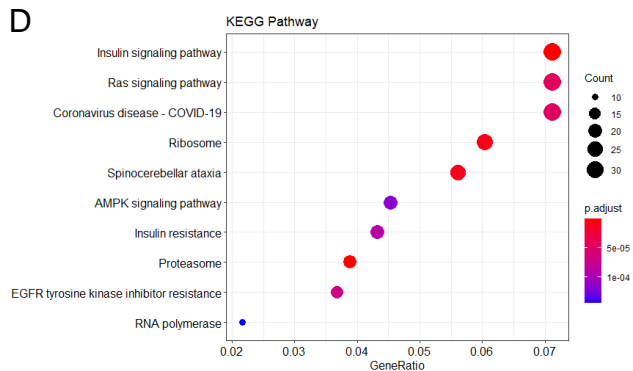

Supplement: Supplementary file 2 — Additional file 2. [file 12866_2021_2392_MOESM2_ESM.pdf]

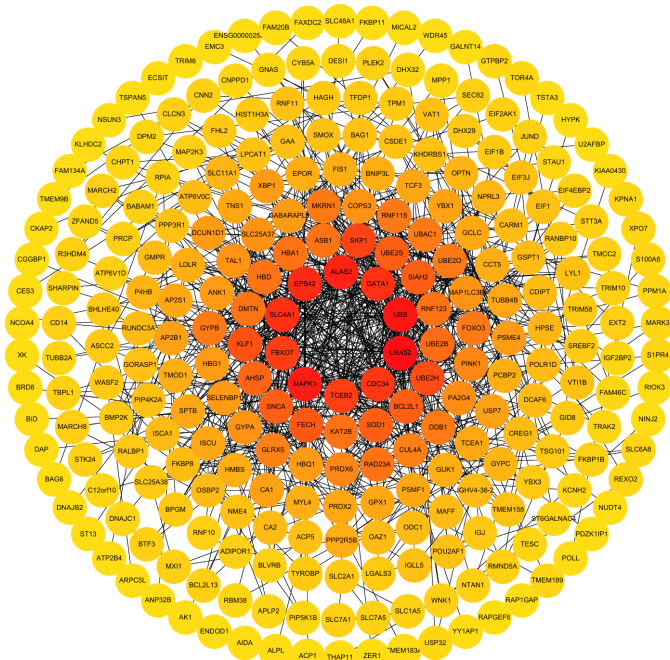

Supplement: Supplementary file 3 — Additional file 3. [file 12866_2021_2392_MOESM3_ESM.pdf]

A

ROC Curve for SKP1

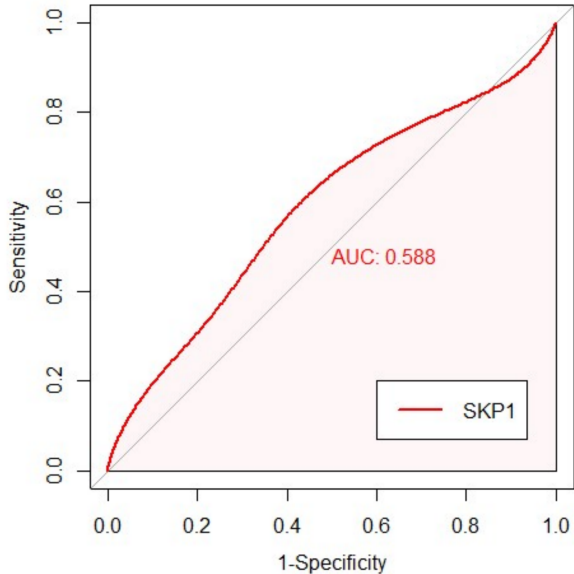

B

ROC Curve for CDC34

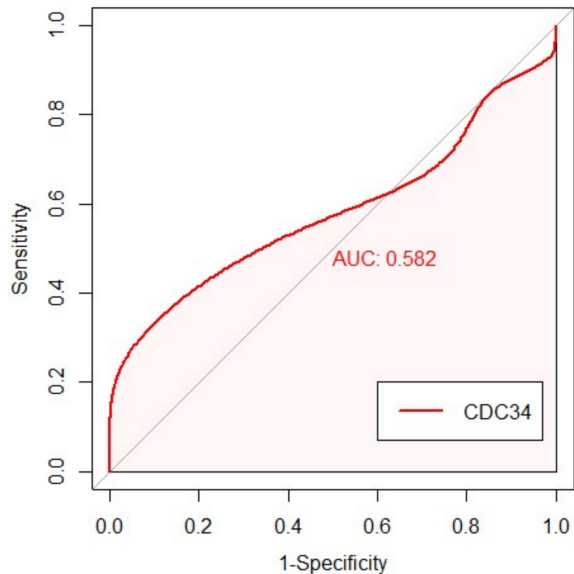

Supplement: Supplementary file 4 — Additional file 4. [file 12866_2021_2392_MOESM4_ESM.pdf]
